# Advances in preclinical evaluation of experimental antibody-drug conjugates 

**DOI:** 10.20517/cdr.2021.37

**Published:** 2021-07-04

**Authors:** Scott K. Lyons, Dennis Plenker, Lloyd C. Trotman

**Affiliations:** Cold Spring Harbor Laboratory, Cold Spring Harbor, NY 11724, USA.

**Keywords:** Antibody-drug conjugate, tumor, preclinical, organoid, CRISPR, preclinical imaging, theranostic

## Abstract

The ability to chemically modify monoclonal antibodies with the attachment of specific functional groups has opened up an enormous range of possibilities for the targeted treatment and diagnosis of cancer in the clinic. As the number of such antibody-based drug candidates has increased, so too has the need for more stringent and robust preclinical evaluation of their *in vivo *performance to maximize the likelihood that time, research effort, and money are only spent developing the most effective and promising candidate molecules for translation to the clinic. Concurrent with the development of antibody-drug conjugate (ADC) technology, several recent advances in preclinical research stand to greatly increase the experimental rigor by which promising candidate molecules can be evaluated. These include advances in preclinical tumor modeling with the development of patient-derived tumor organoid models that far better recapitulate many aspects of the human disease than conventional subcutaneous xenograft models. Such models are amenable to genetic manipulation, which will greatly improve our understanding of the relationship between ADC and antigen and stringently evaluate mechanisms of therapeutic response. Finally, tumor development is often not visible in these *in vivo* models. We discuss how the application of several preclinical molecular imaging techniques will greatly enhance the quality of experimental data, enabling quantitative pre- and post-treatment tumor measurements or the precise assessment of ADCs as effective diagnostics. In our opinion, when taken together, these advances in preclinical cancer research will greatly improve the identification of effective candidate ADC molecules with the best chance of clinical translation and cancer patient benefit.

## INTRODUCTION

In recent years, monoclonal antibodies have become a hugely promising class of anti-cancer therapeutic molecules, given their high affinity and precise specificity for any number of tumor-specific target antigens. As an effective anti-cancer treatment, antibodies have been used to directly and specifically block cell surface receptor signaling (e.g., trastuzumab and Her2 receptor-positive breast cancer^[1]^). Of broader appeal, however, antibodies can be readily modified to carry a variety of treatment or imaging payloads and target them to practically any tumor-specific cell surface antigen. This effectively concentrates the antibody-drug conjugate (ADC) payload to sites of tumor development, significantly reducing possible treatment-related cytotoxicity in other organs or improving the ability to non-invasively image tumors above background noise^[2]^.

The current development pipeline used to translate any promising cancer agent from the bench to the bedside is both high risk and prohibitively expensive in terms of both financial and human cost. The overall percent failure rate of taking any new molecule into the clinic has been reported to exceed 95%^[3]^. The average total development cost to FDA approval has recently been quoted at 2.8 billion USD^[4]^. Recent analysis has suggested that poorly predictive preclinical assays and models are a key contributing factor to this multifaceted problem^[5]^. In addition to the possible misunderstanding of mechanisms of targeting and therapeutic effect, misleading preclinical data also mean that critical “go, not go” decisions are not made until late in the drug development process after considerable research dollars have been spent and people have been enrolled on clinical trials with a weak premise.

As with the clinical development of any anti-cancer agent, certain aspects of ADC performance must first be rigorously evaluated in an experimental preclinical setting. At a minimum, in addition to showing safety, the target tumor antigen should be identified and then rigorously tested to show that *in vivo* accumulation of the ADC is antigen-specific and not the result of off-target interactions or leaky tumor vasculature and the EPR effect (enhanced perfusion and retention)^[6]^. Given that most ADCs in clinical development recognize and bind to human antigens, IHC staining of frozen human tissue microarrays will most likely be preferable over *in vivo* mouse models to predict where appreciable levels of the ADC may accumulate in the human body other than tumor sites. However, the relationship between ADC and target antigen in the context of whole-body physiology and measurements of therapeutic effect and ADC biodistribution can now be interrogated to much higher experimental standards.

We present here several recent advances in preclinical research that stand to significantly raise the rigor by which candidate ADC molecules and anti-cancer drugs can be assessed prior to clinical application. These include the ability to efficiently establish more representative *in vitro* and *in vivo* tumor models from patient-derived material (matching normal, tumor, and metastatic tumor organoid cell lines), the ability to use CRISPR or inducible transgene technology to specifically manipulate the expression of antigen, and advances in non-invasive imaging that allow dynamic tracking of the ADC molecule or resulting treatment effects. Essentially, these advances greatly improve the quality of experimental control, such that the comparisons of ADC accumulation or therapeutic efficacy can be readily made between matched pairs of normal and tumor cells or between matched tumors that only differ in antigen expression. Imaging further permits many of these effects to be seen in the same individual subject dynamically over time, reducing the need for large experimental cohorts. Imaging also enables the standardization of ADC administration based on measured and not assumed tumor parameters, greatly improving the quality of data.

## ADVANCES IN PRECLINICAL CANCER MODELS

The past decade has been transformative for tissue culture technology of patient-derived tumors. Until recently, only a limited number of immortalized 2D cancer cell lines was available to test the preclinical performance of an ADC using xenograft mouse models. Such models remain popular today as they are relatively quick and easy to develop. The cell lines are widely distributed among the research community, and some have been the focus of extensive genomic and gene expression characterization^[7]^. However, such cancer models also have significant deficiencies, can be prone to genetic drift over time, and their ability to accurately model human disease and ultimately predict the clinical performance of candidate therapeutics is questionable. The failure rate of establishing an immortalized tumor cell line in culture by traditional means is extremely high. Human tumors did not evolve to grow on tissue culture plastic as a 2D monolayer; thus, it is questionable how representative the low frequency of successfully established cultures are of the original tumor. Further, although subcutaneous engraftment of such cells *in vivo* is very routine (quick to establish and tumor development is externally visible), such models fail to recapitulate the tumor microenvironment that matches their native tissue of origin.

**Figure 1:**
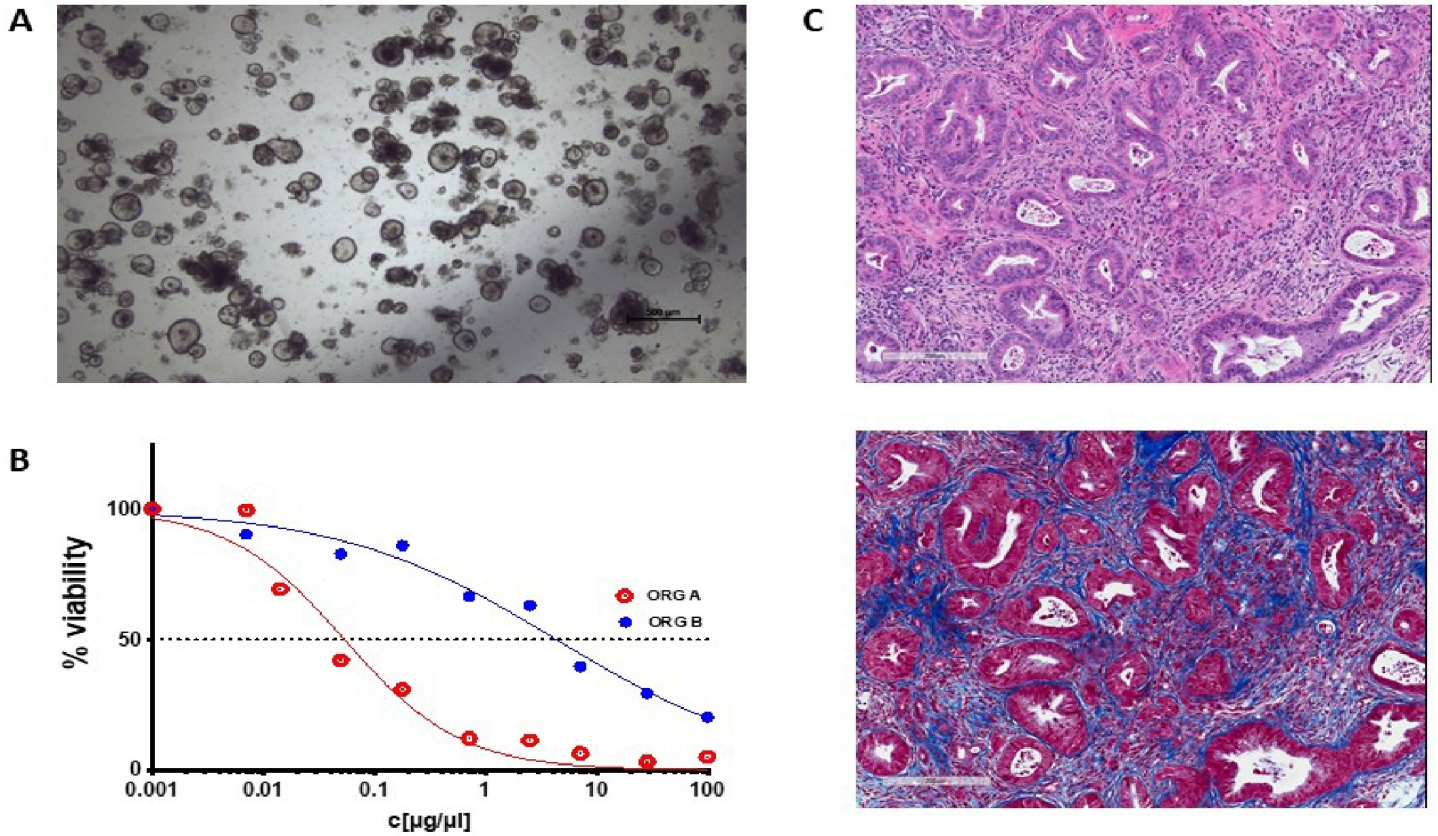
An example of pancreatic tumor organoids growing in culture and *in vivo*. (A) A brightfield microscopy image of organoids growing in structured 3D spheres in Matrigel *in vitro*. Organoids can be efficiently generated from a wide range of epithelial tissues and, as they are genetically stable over an extended time in culture, are amenable to genetic manipulation. (B) A schematic of how an organoid’s sensitivity to a therapeutic can be presented. Organoid cell viability can be measured in a high-throughput manner by standard assay techniques [e.g., CellTiter-Glo (Promega)] and plotted against drug or ADC concentration. The therapeutic responses of normal, tumor, and metastatic organoids derived from the same patient, organoids from different patients, or genetically modified variant organoids can be readily compared. (C) Two serial sections of a pancreatic tumor developed *in vivo *following orthotopic implantation of tumor organoid cells. The top panel is an H&E stain, highlighting regions of tumor cells in deep purple. The bottom panel is a Masson’s trichrome stain, which stains collagen blue. These organoid tumors develop a dense stroma composed of connective tissue and fibroblasts when grown orthotopically *in vivo*, recapitulating key clinical characteristics.

The advent of patient-derived organoid (PDO) cell cultures has set new biologically relevant standards that overcome many limitations of conventional 2D xenograft cell lines^[8]^. Tumor samples received fresh from the operating theatre are processed and plated out *in vitro* in a mixture of growth factors and basement membrane extract such as Matrigel (a viscous matrix resembling a decellularized tissue microenvironment) to provide more natural growth conditions for the cells. As a result, PDOs grown in three dimensions *in vitro* retain cell polarity and some semblance of structure that can provide biologically relevant cues [Figure 1]. The efficiency and general applicability to various tumor types also far surpass that of traditional 2D methods. The success rate of establishing a PDO culture can be as high as 75% for tumors from a wide range of epithelial organs. Once established, they can be propagated *in vitro* with relative ease in defined culture media for extended periods of time. Analyses suggest that organoid cultures are relatively stable genetically for many passages^[9,10]^, which opens up a host of research possibilities regarding their genetic modification and experimental possibilities, as discussed in Sections ii and iii. Another ground-breaking feature of this approach is that it is possible to establish organoid cultures from matching normal, tumor, and metastatic tissue from the same individual. This is of crucial experimental importance given the outbred nature and diverse genetic background of the human population. Moreover, organoids derived from sequential specimens from the same patient have been shown to recapitulate identical sensitivities and resistance to treatment, as observed in the clinic^[11]^.

The Human Cancer Model Initiative (an NIH/NCI-funded project^[12]^) was established as a novel resource to give researchers access to these next-generation models via the American Type Culture Collection. Many of the organoid lines available in this ground-breaking biorepository are also documented with patient and sequencing information. Having such a well-characterized portfolio of cells enables many opportunities for high-quality experimentation to evaluate ADC performance that was impossible or very hard to accomplish in the past. For example, genetically and disease-matched PDOs can be used to assess the cytotoxicity of targeted ADCs *in vitro* or *in vivo* and be compared to matching normal tissue-derived PDOs. As discussed in Section iii, the conjugation of different moieties for imaging can be used to visualize where ADCs accumulate naturally in the body and whether, once bound to the antigen, they stay on the surface of a cell or are internalized.

PDO co-cultures can also be established *in vitro* to examine critical tumor cell interactions with defined aspects of the tumor microenvironment, such as cancer-associated fibroblasts^[13,14]^, and possibly also with immune cells in the near future. Such co-cultures will enable testing of an ADC on tumor and normal cells at the same time, helping to better understand the effects an ADC may have on either cell type or their ability to interact.

The engraftment of human-derived PDO material into a mouse for *in vivo* studies, as with conventional 2D tumor xenograft-based models used for ADC development, necessitates the employment of immune-compromised host strains. As such, such *in vivo* models do not allow the study of tumor interactions with a fully intact host immune system. Further exploration of the important role played by the host microenvironment in PDO tumor development (and potentially ADC interaction) has shown that different implantation sites in the same organ can significantly influence *in vivo* tumor biology. For example, in the case of organoids derived from pancreatic ductal adenocarcinoma (the most common form of pancreatic cancer), it has been shown that the tumor growth pattern and interaction with the microenvironment differ greatly when growing in the parenchyma of the pancreas *vs*. the actual pancreatic duct (where the disease is understood to originate)^[15]^. No one can argue with the relative ease of setting up conventional subcutaneous tumor xenograft models, but studies such as these do raise serious questions regarding what aspects of the disease they accurately recapitulate.

Taken together, these advances in tumor organoid technology enable more complex, rigorous, and informative validation of ADC performance before proceeding to large and expensive clinical studies. The broad diversity of human PDO models available will massively enrich research efforts and possibilities to validate ADC effects in various disease models and thus enable the stratification of the patients who will most likely benefit from treatment with a specific ADC molecule.

## ADVANCES IN GENETIC MANIPULATION

A critical aspect of ADC development is to stringently test and understand the specificity of binding to the target antigen. This knowledge is ultimately key for successful clinical translation and identifying which individual patients are most likely to benefit from treatment with the ADC.

The specificity of the antigen is often demonstrated experimentally using different, non-genetically matched tumor cell lines that differentially express the antigen or a pre-treatment block with non-labeled antibody. These are acceptable methods, but recent advances in genetic manipulation, namely the widespread adoption of CRISPR (clustered regularly interspaced short palindromic repeats), have greatly facilitated the potential adoption of significantly higher experimental standards. 

An ideal experimental scenario to test antigen specificity *in vivo *would be to employ paired preclinical tumor models, identical in every way except for target antigen expression. Tumors plus or minus antigen, developing on contralateral flanks or in matched experimental cohorts, would elegantly demonstrate that observed ADC accumulation at a tumor is specific and not due to off-target interactions or simple passive accumulation via leaky tumor vasculature and the EPR effect.

Traditionally, the only available approach to precisely knock-out the expression of a target gene relied upon homologous recombination between a targeting vector and endogenous allele^[16]^. The approach was technical, inefficient, and time consuming to employ and was seldom performed in any other context than in the targeting of murine ES cells to produce genetically modified mice. The subsequent discovery of RNAi and the ability to easily and specifically knock-down gene expression with shRNA^[17]^ or miRNA^[18]^ was transformative by rapidly facilitating specific and significantly reduced levels of gene expression in practically any eukaryotic cell line of interest. This approach is still valid today, however gene knock-down by RNAi is frequently not 100% and so the target antigen is still expressed to some extent.

The relatively recent discovery, rapid development, and widespread adoption of CRISPR technology has completely revolutionized our ability to precisely modify the genome ^[19]^. Among the many documented applications, it is now relatively straightforward and efficient to generate such matched tumor model pairs, plus or minus the expression of antigen. Briefly, CRISPR introduces precisely targeted double-strand breaks in the genome, which are typically imperfectly repaired by non-homologous end joining. This repair process often results in the microdeletion of one or more nucleotides at the DSB. Accordingly, bi-allelic frame-shifting mutations can be readily introduced into the specific coding sequence of a gene of interest, effectively knocking out its expression.

On occasion, knock-out of gene expression is poorly tolerated by the targeted cell, significantly affecting cell fitness or causing phenotypic drift, such that the genetically paired cell lines are no longer a good match biologically. In those circumstances, inducible transgene technology can be employed (e.g., doxycycline inducible expression^[20]^) to limit the amount of time between antigen knock-out and antibody affinity assay. Further, inducible gene expression can introduce or restore the expression of an antigen in a cell line that the ADC does not otherwise recognize. For example, the expression of a tumor-specific antigen could be readily introduced into a “normal” and non-expressing organoid cell line (as mentioned in Section i); implanted cells in non-induced mice will not express the antigen and so lack affinity for the ADC, whereas induced mice will become antigen positive and show ADC accumulation.

It is significantly easier and faster to manipulate gene expression in cell line-based cancer models than in transgenic mice. Therefore, it is exciting that the advances in tumor modeling discussed above are to some extent tissue-culture based, as this opens up many possibilities for their efficient genetic manipulation and thus thorough and rigorous *in vivo* experimentation.

## ADVANCES IN PRECLINICAL IMAGING

Depending on the nature of the conjugated moiety, ADC molecules can be considered “theranostic”, a term used to describe a molecule with both therapeutic and diagnostic properties. Once a candidate ADC molecule has satisfied stringent *in vitro* and tissue-histology performance criteria, preclinical imaging can be used to dynamically measure both aspects of this, i.e., to visualize the biodistribution of an ADC molecule in the context of the whole body over time and to accurately measure the anticancer effects of ADC treatment. Critically, imaging can be used to standardize the timing of ADC administration (e.g., on the basis of tumor size) across experimental cohorts of mice and can provide meaningful “before and after” ADC treatment measurements of the same tumor in the same individual. Collectively, these experimental advantages serve to reduce the number of assumptions made in an *in vivo* study, greatly improving study robustness and reducing animal cohort size. Non-invasive imaging is also particularly pertinent in the context of the tumor organoid model advances discussed above, as tumors development in orthotopic and deep tissue locations are otherwise not visible externally.

Rather than comprehensively review the field of preclinical imaging, we instead mention here several key imaging practices that we believe are particularly impactful for current and future ADC development.

### Approaches to image antibody biodistribution *in vivo*

ADCs are extremely versatile tumor targeting molecules due to the broad variety of functional groups that can be attached to them, whether therapeutic “warheads” used to treat cancer or diagnostic ones for tumor imaging. All preclinical imaging modalities have relative strengths and weaknesses and so the conjugated imaging moiety should be selected based on the nature of the experimental goal. It should also be noted that different conjugates will influence the *in vivo* biodistribution of an ADC and that not all variant ADC can be presumed to perform equally.

In the preclinical space, the attachment of a fluorescent moiety (e.g., an Alexa Fluor dye^[21]^) to a candidate ADC can uniquely enable direct visualization and accurate quantitation of antibody binding to target cells by microscopy or flow cytometry *in vitro*. However, fluorescence imaging is in general poorly suited for whole-body *in vivo* imaging, as visible wavelengths of light are poorly tissue penetrant and prone to scatter and absorption from overlying tissues^[22]^. Autofluorescence generated from surrounding gram amounts of non-labeled tissue can also prove troublesome in the context of attempting to detect a signal above background from small, milligram-sized tumors. Intravital microscopy, however, is a unique *in vivo *fluorescence-based imaging approach that allows the researcher to look at the accumulation of labeled antibody at the site of tumor development at sub-cellular resolution *in vivo*^[23]^. No other mainstream *in vivo* imaging modality can offer this kind of imaging scale, but it should be noted that IVM only offers a small field of view with limited depth of tissue penetration (0.5 mm) and is only suitable for imaging through an implanted “window” at a single location in the body. Fluorescent immunopeptides are also of additional clinical interest and are being developed for guided intraoperative imaging purposes, to assist surgeons to both locate tumors and set resection margins in theatre^[24]^. We speculate that recent advances in tissue clearing techniques and light sheet microscopy may also enable visualization of fluorescent ADC binding at cellular resolution in reconstructed 3D images of tissue^[25]^.

Optoacoustic (or photoacoustic) imaging is similar in nature to fluorescence-based approaches, but it is better suited for deep tissue imaging. Instead of attaching a fluorophore, this approach relies upon the conjugation of a light absorbing or quenching moiety^[26,27]^, i.e., the attachment of a molecule with a high molecular extinction co-efficient and low quantum yield. Unlike a strong fluorescent label, whereby absorbed excitation energy is largely emitted as red-shifted light, a strong photoacoustic label predominantly emits absorbed excitation energy as heat. When the excitation light is pulsed in nanosecond bursts, the resulting pulses of heat (and associated pulses of thermal tissue expansion) generate an ultrasound signal that can be readily detected. Most imaging modalities offer either high resolution images with poor image sensitivity (e.g., MRI) or highly sensitive and poorly resolved images (e.g., PET). While not yet offering subcellular image resolution similar to fluorescence imaging, optoacoustic imaging is a good compromise whole-body and clinically translatable imaging modality, offering both reasonable imaging resolution and sensitivity at deeper tissue sites.

Arguably the most sensitive and quantifiable way to non-invasively track the biodistribution of an ADC candidate molecule throughout the whole body is by PET (positron emission tomography). Full-size antibodies have a relatively long serum half-life *in vivo* and so conjugation of the positron emitting isotope Zirconium 89 (t_1/2 _= 3.3 days) allows follow up biodistribution scans for more than a week after administration^[28]^. Unlike optical signals, the gamma rays detected by PET are much less prone to scatter or attenuation from overlying tissue. Accordingly, resultant tomographic images are not surface-weighted and allow for the detection and discrimination of individual lesions in relatively close proximity, irrespective of depth in tissue. Derivative antibody fragments, such as Fabs or diabodies, have a significantly shorter serum half-life, often clearing the body within 24 h, and so can be adequately imaged by PET with more conventional and shorter-lived radioisotopes, such as Fluorine 18^[29]^.

In the absence of a formal biodistribution study, whole-body PET images of an antibody can be used to estimate where in the body the same ADC with a different therapeutic radioisotope attached [such as Yttrium 90 (β-emitter) or Actinium 225 (α-emitter)] would accumulate in the body. As mentioned above, ADCs with different conjugated moieties cannot be assumed to behave in an identical fashion *in vivo*. Matched pairs of imaging and diagnostic radioisotopes [such as Scandium 44 (positron emitter for PET imaging) and Scandium 47 (β-emitter for therapy)] are being developed to circumvent such issues, lending greater predictive power to the PET scan^[30]^.

### Advances in imaging the therapeutic effects of an ADC

In addition to being able to non-invasively track the biodistribution of a candidate ADC molecule, preclinical imaging can be equally useful in providing a dynamic and quantifiable measure of tumor cell cytotoxicity and therapeutic effect.

The most obvious preclinical modality to mention in this context is bioluminescence imaging (BLI). For reasons of relative sensitivity, speed, safety (does not involve ionizing radiation), and affordability, this optical imaging modality has been widely adopted by the research community^[31]^. The BLI signal is reliant upon first introducing the expression of a luciferase transgene into the tumor model of interest. An expanding list of candidate enzymes and substrates suitable for BLI have now been described^[32]^; however, enzymes that rely upon ATP to generate light are the most useful for assessing relative tumor cell viability *in vivo*. A highly relevant feature of these enzymes is that dead cells no longer emit light and so the cytotoxic effects of any drug or ADC can be readily assessed following treatment. This viability measure is often possible at experimental time-points far earlier than when gross anatomic changes to the tumor become evident.

**Figure 2:**
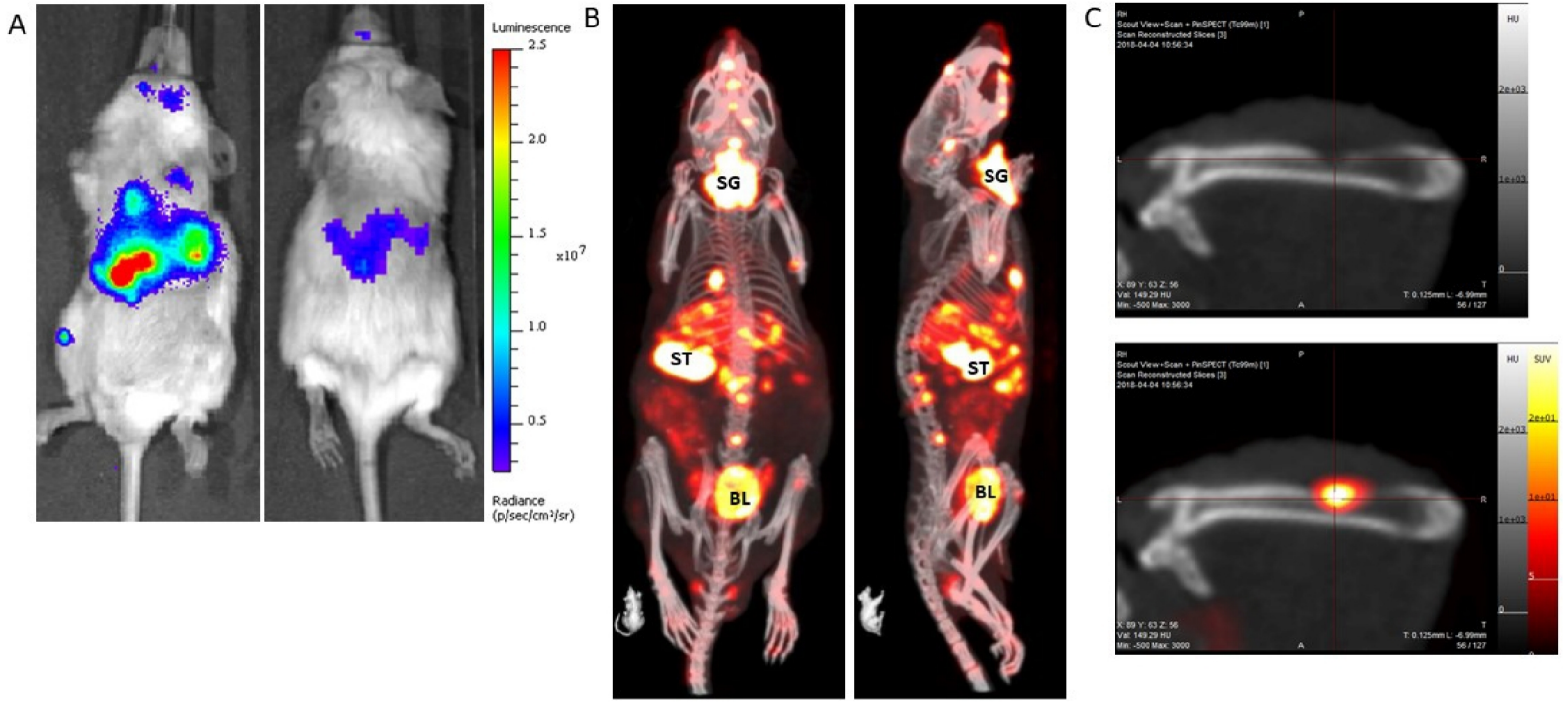
An example of a metastatic prostate cancer model with the tumors labeled with: firefly luciferase (A); and NIS expression (B). The BLI image (A) takes less than 1 min to acquire, and, as only the tumor cells emit light, it clearly shows widespread dissemination of metastases throughout the body, most clearly in the abdomen and leg. The optical signal is absorbed and scattered by overlying tissue, thus it is not possible to resolve individual lesions by this imaging method. A NIS-SPECT image of the same mouse (B) shows the same tumor burden, but now in 3D. Metastases are clearly present and prevalent in the liver and bone (spine, skull, and femur) of this mouse. Some normal organs in the body also express NIS and so also show up on the scan. SG: Salivary gland/thyroid; ST: stomach; BL: bladder (excretion route of ^99m^TcO_4_ probe). (C) Images of a femur from a different mouse with a prostate tumor metastasis. The top panel is a CT image only and shows clear evidence of bone degradation. When the NIS-SPECT image is co-registered, it is clear that an osteolytic lesion is growing in this part of the bone.

We would also like to draw particular attention to a relatively little used imaging technique termed NIS-SPECT. Similar to *in vivo* bioluminescence imaging, whereby cancer cells are first labeled with the expression of a reporter transgene, cells are labeled with Sodium Iodide symporter (NIS) expression^[33]^. Physiologically, NIS expression is predominantly limited to the salivary gland, thyroid, stomach, and lactating breast in the mouse and transports iodine, an essential component of thyroid hormone biosynthesis. Crucially for imaging, NIS also imports the widely available and cheap gamma-emitting radiotracer ^99m^pertechnetate, which can be detected by SPECT. In our experience, NIS-SPECT can readily detect and individually resolve multiple sub-cubic-millimeter-sized metastatic tumor lesions in three-dimensional space (see Figure 2). As background expression of endogenous NIS is effectively absent in all organs other than those mentioned above, the signal-to-noise ratio is generally excellent and this approach can produce visually striking and accurate 3D images of tumor development and metastatic spread *in vivo*. It would be an extremely attractive experimental proposition to co-register the ^89^Zr-labeled ADC biodistribution PET image with the NIS-SPECT tumor image, especially in the context of metastatic disease models that have been rendered positive or negative for expression of the target antigen (as mentioned in Section ii).

## CONCLUDING REMARKS

In the immediate future, it is exciting to consider that the confluence of advances in cancer modeling, genetic manipulation and preclinical imaging discussed here will be able to provide significantly more robust evaluation of candidate ADC performance prior to clinical application. This is especially pertinent given the high attrition rate of promising candidate molecules as they progressively develop from the laboratory to the clinic. For practical reasons, we are not suggesting that these new techniques completely replace existing experimental approaches, but they should be considered and applied to some extent to rigorously explore key ADC development milestones. Improvements in the methods employed to more stringently test and assess the *in vivo* performance of any candidate drug or ADC can only stand to enable more informed “go, no go” decisions earlier in their development and ultimately ensure that efforts and resources are focused on those candidate molecules identified as most likely to succeed in the clinic and benefit cancer patients.

## DECLARATIONS

### Copyright

© The Author(s) 2021.
